# Clinical impact of the use of chronic suppressive antibiotics against recurrent ventricular assist device infections

**DOI:** 10.1128/spectrum.00398-24

**Published:** 2024-10-04

**Authors:** Shinya Yamamoto, Koh Okamoto, Hiraku Kumamaru, Makoto Saito, Hiroshi Ito, Marie Yamashita, Yoshiaki Kanno, Mahoko Ikeda, Sohei Harada, Shu Okugwa, Mitsutoshi Kimura, Osamu Kinoshita, Minoru Ono, Takeya Tsutsumi, Kyoji Moriya

**Affiliations:** 1 Department of Infectious Diseases, The University of Tokyo Hospital, Tokyo, Japan; 2 Department of Healthcare Quality Assessment, Graduate School of Medicine, University of Tokyo, Tokyo, Japan; 3 Division of Infectious Diseases, Advanced Clinical Research Center, Institute of Medical Science, University of Tokyo, Tokyo, Japan; 4 Department of Infection Control and Prevention, The University of Tokyo Hospital, Tokyo, Japan; 5 Department of Cardiac Surgery, The University of Tokyo Hospital, Tokyo, Japan; NHLS Tygerberg/Stellenbosch University, Cape Town, Western Cape, South Africa

**Keywords:** ventricular assist devices, chronic suppressive antibiotics

## Abstract

Ventricular assist device (VAD) infections are frequent causes of hospital readmission. The risk factors and optimal preventive strategies for such, including chronic suppressive antibiotics (CSA), remain uncertain. We performed a single-center, retrospective, observational cohort study assessing continuous flow VAD recipients who underwent implantation between 2008 and 2018 in Japan. From primary VAD infection (VADI), we followed the patients for recurrent infection, defined as relapsing VAD-specific (e.g., localized infections) or VAD-related (e.g., bacteremia) infections requiring hospital readmission. CSA was defined as the use of oral antimicrobial agents continued beyond initial antibiotic use until transplantation, VAD withdrawal, VADI recurrence, or death. Survival analysis was performed to identify risk factors for recurrent infection accounting for competing risks (e.g., deaths and transplants). Among 163 eligible patients, 76 patients had VADIs. The main causative organism in primary VADI was *Staphylococcus aureus* (63%, 48/76). Among them, 41 had recurrent infections, whereas 35 had none during the follow-up period (median, 335 days). Thirty-six patients received CSA for a median of 478 days. Although CSA was associated with a decreased risk of recurrent infection [adjusted sub-distribution hazard ratio (SHR), 0.40; 95% confidence interval (CI), 0.18–0.89; *P =* 0.03], this protective effect was observed only after primary VAD-specific infection (SHR, 0.28; 95% CI, 0.12–0.64; *P* < 0.01) but not after VAD-related infection. Surgical procedures during primary VADI were associated with an increased risk (SHR, 2.00; 95% CI, 1.10–3.66; *P* = 0.02). One patient had an adverse drug reaction. CSA may be an effective approach to limit relapsing VADIs following a primary VAD-specific infection with minimal adverse events.

**IMPORTANCE:**

Ventricular assist device infections (VADIs) are a significant complication leading to hospital readmissions. However, the risk factors and optimal preventive strategies for VADI remain unclear. This study investigated the effectiveness of chronic suppressive antibiotic therapy in patients with VADI. We found that the use of chronic suppressive antibiotic therapy was associated with a reduction in the risk of VADI recurrence with few adverse reactions. Our findings suggest the potential benefit of chronic suppressive antibiotics in preventing infections in selected cases. Our findings are relevant for the management of patients with ventricular assist devices awaiting heart transplantation, providing valuable insights for clinical practice.

## INTRODUCTION

A ventricular assist device (VAD) is a cornerstone of therapy for end-stage heart failure as a bridge to heart transplantation or destination therapy (1, 2). Because of the limited availability of heart transplant donors (3), there has been a steady increase in the number of VAD implantations as well as the time on VAD for each VAD recipient (4). Infectious complications (VAD infection; VADI) are one of the major complications among VAD recipients. They occur in 22.6%–60% of recipients (5, 6) and are a frequent cause of hospital readmission (7, 8). Furthermore, VADI is an independent risk factor for cerebral emboli and death (9). Importantly, VADI often recurs. Previous studies have revealed that up to 85% of patients experience recurrence (7, 10, 11). Moreover, our previous study has shown that VADI was the most common reason (36%) of hospital readmission (7); therefore, prevention of recurrent VADIs is of paramount importance.

Multiple strategies have been proposed to prevent primary VADI, including patient education, perioperative prophylaxis, driveline care, and chronic suppressive antibiotics (CSA) (6). However, only a few studies with varying methods and outcomes have focused on the role of CSA in VADI prevention, and no firm conclusions have been reached thus far. The 2019 American Society of Transplantation Infectious Diseases Community Practice Guidelines offered no recommendations on CSA for VADI and only discussed the benefits and risks of CSA (6).

CSA is used as a suppressive therapy for a variety of hardware infections, including spinal hardware infections, prosthetic joint infections, and vascular graft infections when the hardware cannot be removed surgically (12–15). In this study, we hypothesized CSA might reduce the incidence of recurrent VADI, thereby reducing the frequency of hospital readmission.

## MATERIALS AND METHODS

### Study design, setting, and participants

This single-center, retrospective observational study was performed at the University of Tokyo Hospital (UTH), a 1,217-bed tertiary-care teaching hospital in Tokyo, Japan. All adults aged ≥18 years who underwent continuous flow (CF) VAD implantation as a bridge to heart transplantation using HeartMate II (Abbott, USA), Jarvik 2000 (Jarvik Heart Inc., USA), DuraHeart (Terumo Heart, Japan), EVAHEART (Sun Medical Technology Research Corp, Japan) between April 2008 and March 2018, and had subsequent VAD infections requiring hospital admission, were included. Patients with extracorporeal VAD and those who died during the primary VADI episode were excluded from the study. When a patient underwent placement of VAD multiple times, for instance, an extracorporeal followed by implantable VAD, the first placement of an implantable VAD was regarded as the index implantation. The institutional review board approved this study (approval number: 2020159NI), and the requirement for informed consent was waived due to the retrospective observational nature of the study.

### Microbiological analysis

The samples were collected based on the judgment of the attending physician. Identification was performed using matrix-assisted laser desorption/ionization time-of-flight mass spectrometry (Biotyper, Bruker Daltonics, Bremen, Germany). Antimicrobial susceptibility testing was performed using MicroScan WalkAway system (Beckman Coulter, Brea, CA, USA), and the results were assessed following the Clinical and Laboratory Standards Institute guidelines M100 28th edition (16).

### Definitions

VADI was defined according to the International Society for Heart and Lung Transplantation guidelines (17) where infectious complications of VAD are largely classified as VAD-specific infections, VAD-related infections, and non-VAD infections (17). In this study “VADI” included both VAD-specific and VAD-related infections. These definitions were also used in previous studies (10, 18). We defined recurrent VADI as VAD-specific or VAD-related infection, regardless of culture negativity, that required hospital readmission for surgical management or intravenous antibiotic therapy, excluding minor recurrent VADIs that were managed in outpatient settings. Surgical procedures included debridement of the driveline, pocket, or mediastinum, revision, and VAD pump change for source control. We defined initial therapy as intravenous antibiotic use for primary VADI. We defined CSA as the use of oral antimicrobial agents that are susceptible to the microorganisms causing primary VADI, continued beyond after the completion of the initial course of intravenous antibiotic use for the primary VADI until transplantation, VAD withdrawal, VADI recurrence, or death. The decision to start CSA as well as the selection of the agent was at the discretion of the treating physician.

### Data collection

Data were collected via a manual chart review. Microbiological data for cultures from blood or samples related to VAD (i.e., drainage fluid from the VAD pocket) were retrieved from the microbiology database.

### Statistical analysis

Competing risk events survival analysis was conducted (19). The observation period began after the initial intravenous antibiotic treatment, and the first recurrence was the outcome. Death, heart transplantation, and VAD removal were handled as competing risk events (Fig. S1). The observation period was censored at the time of hospital transfer or on 3 March 2020. Cumulative incidence function (CIF) was used to describe the proportion of patients who had recurrent VADI. Pepe and Mori test was performed to analyze CIFs of recurrent VADI between patients who received CSA and those who did not. We fitted Fine and Gray’s sub-distribution hazard model to identify factors predicting recurrent VADI. Use of CSA, type of infection, presence of a cardiac implantable electronic device, the causative organism for the primary VADI, and long-term VAD use without surgical procedure were reported features in patients with recurrent VADIs (10, 11, 20, 21) and were therefore included in the final multivariable model. A separate multivariable model including an interaction term between CSA use and type of infection was conducted to assess the effect modification. Huber-White sandwich estimator of variance was used to obtain robust standard errors. All analyses were performed using the Easy R version R-3.6.3 (22), JMP Pro 16 (SAS Institute, Japan), and Stata 16 MP (StataCorp, USA).

## RESULTS

Among 163 patients who received CF-VAD implantation between April 2008 and March 2018 at the UTH, 76 (47%) patients had at least one VADI requiring hospital admission. The median age at the primary VADI was 41 years [interquartile range (IQR), 34–50] (Table 1). Eighteen patients (24%) were female. The underlying etiology of advanced heart failure was predominantly dilated cardiomyopathy (57 patients, 75%). HeartMate II was implanted in 31, EVAHEART in 15, Jarvik 2000 in 17, and DuraHeart in 13. Sixty-three patients (82%) had a VAD-specific primary infection (5 pump/cannula/pocket infections and 58 superficial or deep driveline infections), whereas 13 patients had a VAD-related primary infection [11 blood stream infections (BSIs) and 2 mediastinitis].

**TABLE 1:** Comparison of clinical characteristics of 76 continuous flow ventricular assist device recipients by use of chronic suppressive antibiotics

	Total (*N* ＝ 76）	With CSA (*N* = 36)	Without CSA (*N* = 40)	*P* value
Clinical characteristics				
Age, years^*a*^	41 (34–50)	44 (37–54)	40 (28–50)	0.08
Male sex	58 (76%)	28 (78%)	30 (75%)	0.79
Body mass index^*a*^	21 (18–24)	21 (18–23)	22 (19–25)	0.42
Alcohol use	7 (9%)	3 (8%)	4 (10%)	1.00
Tobacco use	23 (30%)	11 (31%)	12 (30%)	1.00
Etiology of heart failure				0.46
Ischemic cardiomyopathy	7 (9%)	2 (6%)	5 (13%)	
Dilated cardiomyopathy	57 (75%)	27 (75%)	30 (75%)	
Others	12 (16%)	7 (19%)	5 (13%)	
Type of VAD				0.09
HeartMate II	31 (41%)	19 (53%)	12 (30%)	
Jarvik 2000	17 (22%)	9 (25%)	8 (20%)	
DuraHeart	13 (17%)	4 (11%)	9 (23%)	
EVAHEART	15 (20%)	4 (11%)	11 (28%)	
Previous cardiac surgery	34 (44%)	19 (53%)	15 (38%)	0.25
Cardiovascular implantable electronic device	36 (47%)	19 (53%)	17 (43%)	0.49
Chronic kidney diseases and hemodialysis	9 (12%)	2 (6%)	7 (18%)	0.16
Diabetes mellitus	5 (7%)	2 (6%)	3 (8%)	1.00
Initial VAD infection				
Time from implantation to initial infection, days^*a*^	332 (75–534)	368 (60–809)	303 (162–403)	0.54
Classification of infection				0.55
VAD-specific infection	63 (82%)	31 (86%)	32 (80%)	
Driveline infection	58	29	29	
Pump or cannula or pocket infection	5	2	3	
VAD-related infection	13 (17%)	5 (14%)	8 (20%)	
Blood stream infection	11	4	7	
Mediastinitis	2	1	1	
Microbiology				<0.001
*Staphylococcus aureus*	48 (63%)	32 (88%)	16 (40%)	
*Pseudomonas aeruginosa*	15 (20%)	1 (3%)	14 (35%)	
Others	13 (17%)	3 (8%)	10 (25%)	
Surgical procedure at the time of initial infection	24 (32%)	12 (33%)	12 (30%)	0.81
Duration of initial therapy (days)	23.5 (13-43)	21 (10–43)	24 (16–48)	0.45

^*a*^ Median (IQR).

The median time from implantation to the primary VADI was 332 days (IQR, 75–534). Twenty-four patients underwent surgery at the time of primary VADI. Eighteen patients had driveline-related incisions and drainage, one patient had a pump change, three patients had exchange CF-VAD, and two patients underwent surgery for mediastinitis. The median duration of initial therapy with intravenous antibiotics for primary VADI was 23.5 days (IQR, 13–43). Thirty-six patients (47%) received CSA for a median of 478 days (IRQ, 224–672), mostly cefaclor (23 patients, 63%) or trimethoprim-sulfamethoxazole (nine patients, 25%). Most CSA (92%, 33/36) were started immediately following the initial therapy (Fig. S2).

**Fig 1:**
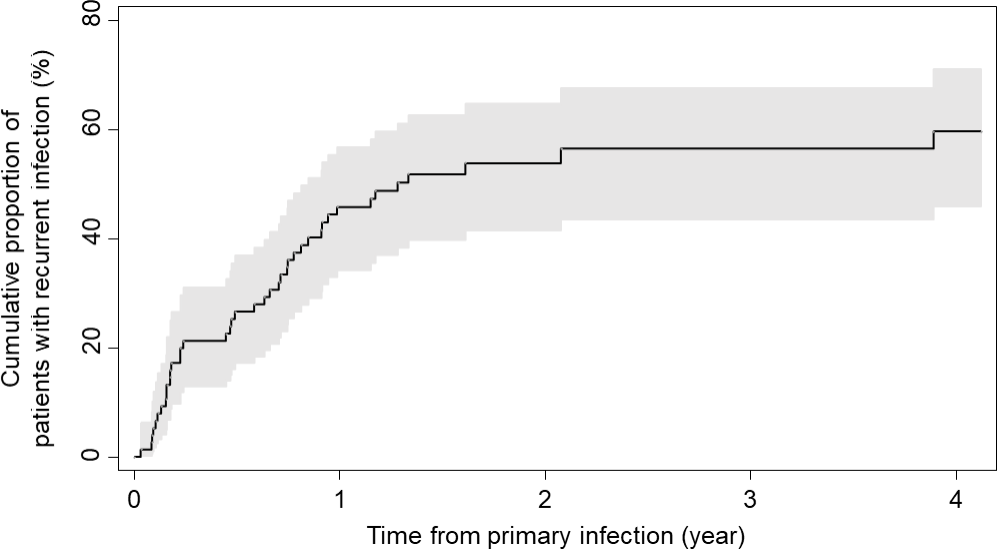
Cumulative incidence function of recurrent ventricular assist device infection for all cases. Shaded area represents the confidence interval around the curve.

During a median follow-up of 335 days (IQR, 127–600) after the primary VADI, 41 of the 76 patients [CIF, 60%; 95% confidence interval (CI), 46%–71%] had recurrent VADI (31 driveline infections; 4 pump, cannula, or pocket infections; 6 BSIs) (Table 2). The median time from the primary VADI to the recurrent VADI was 214 days (IQR, 61–334). A total of 46% of patients had recurrence within a year from the primary VADI, including 21% within 100 days, and further 14% after 1 year (Fig. 1).

**TABLE 2:** Comparison of clinical outcome events of VADI with or without CSA^*a*^

Outcome events	Total (*N* = 76)	With CSA (*N* = 36)	Without CSA (*N* = 40)	*P* value
Recurrent VADI episode	41 (54%)	13 (36%)	28 (70%)	0.005
All-cause death	11 (14%)	5 (14%)	6 (15%)	1.00
Infection-related death	1 (1%)	1 (3%)	0	0.47

^*a*^ Median (interquartile range).

In the primary VADI, *S. aureus* accounted for 63% (48/76 patients; 41 methicillin-susceptible and 7 methicillin-resistant), and *Pseudomonas aeruginosa* accounted for 20% (15/76 patients) (Table 1; Table S1). The causative organisms of the 11 initial BSI were *S. aureus* (*N* = 7), *Staphylococcus epidermidis* (*N* = 1), *P. aeruginosa* (*N* = 1), *Serratia marcescens* (*N* = 1), and *Enterobacter asburiae* (*N* = 1). Among 41 patients who had recurrent VADI, 25 (61%) recurrent episodes were relapses caused by the same organism as the primary VADI (*S. aureus* in 17 patients, *P. aeruginosa* in 8 patients). The remaining 15 patients (37%) had reinfection with other organisms (Table S1). Methicillin-resistant *S. aureus* was found in three patients, and quinolone-resistant and carbapenem-resistant *P. aeruginosa* was found in one patient with recurrent episodes (Fig. S3).

**Fig 2:**
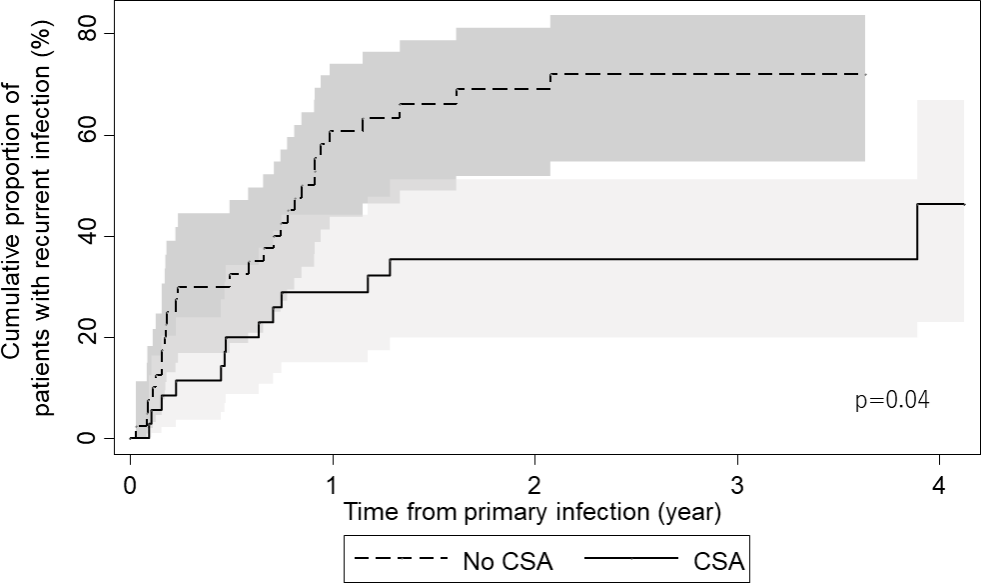
Cumulative incidence function of recurrent ventricular assist device infection for with or without CSA cases. The shaded area represents 95% confidence intervals. *P* value is derived from Pepe and Mori test.

Next, to examine the effect of CSA, we divided the patients into two groups: those with and without CSA. The clinical characteristics of the patients with and without CSA were mostly similar (Table 1). The recurrence rate (CIF) at 365 days after the primary VADI was 29% (95% CI 15%–44%) in the CSA group and 61% (95% CI 44%–74%) in the non-CSA group (Fig. 2). Competing risk events analysis of recurrent VADI using Fine and Gray’s sub-distribution hazard model revealed that CSA was associated with a decreased risk of recurrent VADI [adjusted sub-distribution hazard ratio (SHR), 0.40; 95% CI, 0.18–0.89; *P* = 0.03], whereas surgical intervention for primary VADI was associated with an increased risk (adjusted SHR, 2.16; 95% CI, 1.15–4.03; *P* = 0.02) (Table 3). When the impact of CSA was assessed for each type of the primary infection, CSA was associated with a decreased risk of recurrent VADI among those with VAD-specific primary infection (adjusted SHR, 0.28; 95% CI, 0.12–0.64; *P* < 0.01), whereas it was not among those with VAD-related primary infection (adjusted SHR, 2.08; 95% CI, 0.38–11.36; *P* = 0.40) (Table 4).

**TABLE 3:** Competing risk events analysis of recurrent VADI using Fine and Gray’s sub-distribution hazard model^*a*^

Variable	Observed proportion of events	Univariable		Multivariable	
		Unadjusted SHR (95% CI)	*P* value	Adjusted SHR (95% CI)	*P* value
Chronic suppressive antibiotics	36% (13/36) vs 70% (28/40)	0.43 (0.22–0.82)	0.01	0.40 (0.18–0.89)	0.03
Cardiovascular implantable electronic device	58% (21/36) vs 50% (20/40)	1.09 (0.59–2.00)	0.79	1.21 (0.62–2.37)	0.58
Causative pathogen					
*S. aureus*	50% (24/48)	Reference		Reference	
*P. aeruginosa*	80% (12/15)	2.13 (1.05–4.32)	0.04	1.36 (0.58–3.17)	0.48
Others	38% (5/13)	0.61 (0.25–1.49)	0.28	0.38 (0.13–1.13)	0.08
Surgical intervention for primary infection	71% (17/24) vs 46% (24/52)	1.89 (1.03–3.47)	0.04	2.16 (1.15–4.03)	0.02
VAD-related infection (compared with VAD-specific)	46% (6/13) vs 55% (35/63)	0.78 (0.31–1.96)	0.60	1.07 (0.35–3.34)	0.90
Age (years)	40 (41) vs 42 (35)	0.99 (0.97–1.01)	0.44		
BMI (kg/m^2^)	21.2 (41) vs 21.5 (34)	0.96 (0.88–1.05)	0.40		
Male	53% (31/58) vs 55% (10/18)	0.88 (0.43–1.78)	0.72		
Diabetes	60% (3/5) vs 53% (38/71)	1.16 (0.40–3.34)	0.78		
CRD	55% (5/9) vs 53% (36/67)	0.94 (0.36–2.42)	0.90		
Previous cardiac surgery	58% (20/34) vs 50% (21/42)	1.23 (0.67–2.25)	0.50		
Alcohol	57% (4/7) vs 53% (37/69)	0.91 (0.35–2.39)	0.86		
Smoking	52% (12/23) vs 54% (29/53)	0.90 (0.47–1.74)	0.76		

^*a*^ BMI, body mass index; CRD, chronic renal disease.

**TABLE 4:** Competing risk events analysis of recurrent VADI with interaction between primary infection type and CSA^*b*^

	Multivariable	
Variable	Adjusted SHR (95% CI)	*P* value
Chronic suppressive antibiotics^*a*^		
VAD-specific infection	0.28 (0.12–0.64)	0.003
VAD-related infection	2.08 (0.38–11.36)	0.40
VAD-related infection (compared with VAD-specific)^*a*^		
With chronic suppressive antibiotics	0.58 (0.15–2.21)	0.42
Without chronic suppressive antibiotics	4.33 (0.96–19.55)	0.06
Cardiovascular implantable electronic device	1.23 (0.64–2.34)	0.53
Causative pathogen		
*S. aureus*	Reference	
*P. aeruginosa*	1.17 (0.52–2.64)	0.71
Others	0.31 (0.11–0.87)	0.03
Surgical intervention for primary infection	2.00 (1.10–3.66)	0.02

^*a*^ Stratum-specific adjusted SHRs are shown.

^*b*^ *P* value for interaction is 0.03.

Among those who received CSA, only one patient (2%) had a significant adverse drug reaction (pancytopenia) due to trimethoprim-sulfamethoxazole. None of the other patients required discontinuation or change in the antibiotic regimen due to adverse drug reactions. *Clostridioides difficile* infection and recurrent VADI due to new multidrug-resistant bacteria did not occur.

## DISCUSSION

In this single-center retrospective study of VAD recipients who had been on the waiting list for heart transplant for years, 47% of VAD recipients experienced VADI, and 46% of VADI patients experienced recurrence within 1 year. We found that CSA after primary VADI was associated with a decreased risk of hospital readmission due to recurrent VADI particularly when the primary infection was VAD-specific (i.e., localized) infection, without major adverse drug reactions. In addition, the surgical procedure at the time of primary VADI was associated with an increased risk of recurrent VADI.

The incidence of recurrent VADI is highly variable, ranging from 13% to 85%, depending on the center and length of observation (7, 10, 11). One study revealed that recurrence occurred after a mean of 285 (range, 43–1,055) days (10), and *S. aureus* was the most common microbiological agent (10, 11). These characteristics are consistent with those of our cohort.

The role of the CSA in VADI remains controversial. No randomized controlled trials have addressed this issue, and a few retrospective observational studies, mostly single-center studies, have shown mixed results (10, 18, 23–26). Some reported that CSA was effective in preventing VADI recurrence/relapse. Simon et al. reviewed 35 pulsatile VAD (Heartmate, Thoratec) recipients with VADI, predominantly BSIs, and found that patients who received continued antibiotic therapy beyond 6 weeks had a lower risk of recurrent VADI compared to those who did not (10% vs 71%) (25). Notably, *S. aureus* infections appeared to be associated with a higher risk of recurrence and thus likely required continued antibiotic therapy. Similarly, Garrigos et al. reported that CSA was effective in preventing VADI recurrence/relapse (27).

In contrast, others reported that the CSA was not effective in preventing relapse or recurrence of VADIs. Stulak et al. evaluated the role of chronic antibiotic therapy for the primary prophylaxis of driveline infections (DLIs) in 285 recipients of CF-VAD (HeartMate II) at two institutions and found no significant difference in the incidence of DLIs between patients who did and did not receive CSA (23). Moreover, a single-center, retrospective study (*n* = 69) by Hamad et al. found no difference between with CSA and without CSA groups (44% vs 38%) in the proportion of DLI relapse due to same organism (28). Notably, these studies investigated the incidence of recurrence itself whereas we focused on hospital readmission due to disease recurrence.

In our study, the CSA therapy was associated with a 65% reduction in the risk of recurrent VADIs requiring hospital readmission, although this protective effect was observed only when the primary VADI was VAD-specific infection (DLI and pump infection). Furthermore, the development of resistant organisms and adverse drug reactions, which are major potential drawbacks of CSA therapy, were uncommon in our study, where cefaclor was most used for methicillin-susceptible *S. aureus* VADIs. The results of our study would be an important addition to existing data, given the magnitude of the protective effect, the size of our cohort, and the duration of follow-up. Our findings suggest that CSA therapy has the potential to delay and reduce recurrent VADI requiring hospitalization. However, this strategy should be cautiously applied in carefully selected patients until relevant questions, such as the indication (i.e., causative organisms), optimal duration of therapy, optimal agent, and potential for selection of resistant organisms are further clarified.

The role of source control in VADI remains uncertain. One study reported that 22.4% of the patients underwent surgical procedures after primary VADIs (29). In our study, the surgical procedure at the time of the primary VADI was associated with an increased risk of recurrence in multivariable analysis. There are two possible explanations for this observation. First, patients requiring a surgical procedure themselves might have been at an increased risk for VADI by being more severely ill, as seen in other studies (10, 29). However, most of our patients had local infections and only four patients received VAD/pump changes. Second, the difficulty in complete eradication of microorganisms with surgery might have resulted in recurrent VADI. Indeed, in our study, 63% of patients were infected with the same bacterial pathogen in the recurrent episode as was seen in the first episode. However, the median time of recurrent episodes appears to be too long to detect recurrence due to inadequate surgical therapy. Nonetheless, our findings require further investigation.

We acknowledge several limitations of our study. First, our study is subject to shortcomings due to the nature of this single-center retrospective observational design, including information bias and confounding by potential unmeasured factors. Especially, CSA was given at the discretion of the treating physician, and minor practice changes may have occurred during the 10-year study period. While we collected and adjusted for important known determinants of VADI recurrence, there may be residual confounding. Second, our findings must be interpreted with caution if directly applicable to other institutions given the scope and clinical characteristics of participants and setting of our study. We defined the recurrence as hospital readmission due to VADI; the indication for hospitalization was at the discretion of the attending physician. The decision to admit may have been affected by factors other than the severity of infection, and the indication for hospitalization may vary according to geographical location and health systems. Moreover, the role of CSA in minor recurrent VADIs treated in outpatient is yet to be explored. Notably, the majority of VADI in our patients were local infections caused by methicillin-susceptible *S. aureus*, which allowed for more options for antibiotic selection. Our findings might not be applicable to those with VADI, predominantly BSI or VADI, caused by organisms with limited oral antibiotic options. Similarly, indication, selection, and details of surgical procedures performed for VADI may differ depending on local practice, and the role of surgical procedures should be explored further in future studies. Lastly, in our study, recurrent VADI mostly occurred within 1 year after the completion of initial therapy, and we could not assess if the duration of CSA had affected the outcome. Therefore, the optimal duration of CSA merits further research.

In conclusion, VADI and recurrent VADI were both common among VAD recipients. CSA after the initial intravenous antibiotic therapy may reduce the risk of hospital readmission due to recurrent VADI without major side effects if organisms are susceptible to antibiotics. Our findings may have important implications for VAD recipients who have a VAD-specific infection as a primary infection to reduce the risk of recurrent VADI, in view of the increasing and prolonging use of VAD support worldwide.

## SUPPLEMENTAL MATERIAL

Supplemental material spectrum.00398-24-s0001.pdf Fig. S1 to S3; Table S1.

ASM does not own the copyrights to Supplemental Material that may be linked to, or accessed through, an article. The authors have granted ASM a non-exclusive, world-wide license to publish the Supplemental Material files. Please contact the corresponding author directly for reuse.
