# Supplementary material for: Clinical impact of the use of chronic suppressive antibiotics against recurrent ventricular assist device infections
Source: Microbiol Spectr. 2024 Oct 4;12(11):e00398-24. doi: 10.1128/spectrum.00398-24 (PMC11537063; doi:10.1128/spectrum.00398-24)

Supplementary Table and Figure Legends

Supplementary Table 1. List of microorganisms and chronic suppressive antibiotics in ventricular assist device infection

CNS: coagulase negative staphylococci, MSSA: methicillin susceptible Staphylococcus aureus, MRSE: methicillin resistant Staphylococcus epidermidis, MRSA: methicillin resistant Staphylococcus aureus, MDRP: multidrug resistant Pseudomonas aeruginosa, ESBL: extended spectrum beta lactamase.

ST: sulfamethoxazole/trimethoprim, CCL: cefaclor, LVFX: levofloxacin.

VAD: ventricular assist device, VADI: ventricular assist device infection, CSA: chronic suppressive antibiotics.

a CSA was started with ST and RFP, and was later changed to RFP alone at treating physician's discretion.

Supplementary Figure 1. Time flow of the observation period of VADI in this study.

VAD: Ventricular assist device

VADI: Ventricular assist device infection

CSA: Chronic suppressive antibiotics

Supplementary Figure 2. Summary of all ventricular assist device infection cases with or without chronic suppressive antibiotics.

CSA: Chronic suppressive antibiotics

Supplementary Figure 3. Breakdown of causative microorganisms in second ventricular assist device infection cases

MSSA: methicillin susceptible Staphylococcus aureus

MRSA: methicillin resistant Staphylococcus aureus

P.aeruginosa: Pseudomonas aeruginosa

ST: sulfamethoxazole/trimethoprim

VADI: Ventricular assist device infection

CSA: Chronic suppressive antibiotics

Supplementary Table 1. List of microorganisms and chronic suppressive antibiotics in ventricular assist device infection

First VADI	CSA	Recurrent VADI
CNS	ST	No recurrence
MRSE	ST	MSSA
MSSA	CCL	No recurrence
Serratia marcescens	ST (ineffective)	No recurrence
MSSA	CCL	No recurrence
MSSA	ST	No recurrence
MRSE	Minocycline	No recurrence
MRSA	Rifampicin [a]	No recurrence
MSSA	CCL	No recurrence
MSSA	CCL	No recurrence
MSSA	CCL	No recurrence
MSSA	CCL	No recurrence
MSSA	CCL	No recurrence
MSSA	Amoxicillin	No recurrence
MSSA	CCL	No recurrence
MSSA	CCL	No recurrence
MSSA	CCL	No recurrence
MRSA	ST	No recurrence
MSSA	CCL	No recurrence
MSSA	CCL	*Staphylococcus hominis, capitis*
MSSA	CCL	MSSA
MSSA	CCL	No recurrence
MRSA	ST	No recurrence
MSSA	CCL	No recurrence
Citrobacter species	CCL (ineffective)	*Peptostreptococcus* species
MSSA	CCL	No recurrence
MRSA	ST	MRSA
MSSA	CCL	MSSA

MRSA	ST	MRSA
MSSA	CCL	No recurrence
MRSA	ST	MRSA
MSSA	CCL	MSSA
MSSA	CCL and LVFX	*Pseudomonas aeruginosa*
MRSA	ST	*Pseudomonas aeruginosa*
Pseudomonas aeruginosa	ST (ineffective)	*Pseudomonas aeruginosa*
MSSA	CCL	MSSA
MSSA	CCL	No recurrence
Pseudomonas aeruginosa	LVFX	*Pseudomonas aeruginosa*
MSSA	CCL	MSSA
MSSA		No recurrence
Citrobactor koseri		No recurrence
MSSA		No recurrence
CNS		No recurrence
MSSA		MSSA
Pseudomonas aeruginosa		No recurrence
Pseudomonas aeruginosa		*Pseudomonas aeruginosa*
Entrobacter asburiae		No recurrence
MSSA		Recurrence (no organism identified)
MRSE		MSSA
MDRP		MDRP
Corynebacterium species		No recurrence
MSSA		MSSA
Pseudomonas aeruginosa		No recurrence
Pseudomonas aeruginosa		*Pseudomonas aeruginosa*
Pseudomonas aeruginosa		*Enterobacter asburiae*
MSSA		*Streptococccus sanguinis*
MRSE		No recurrence
Citrobacter koseri		Recurrence (no organism identified)
Pseudomonas aeruginosa quinolone resistant		ESBL producing *Escherichia coli*

MSSA		*Pseudomonas aeruginosa*
MSSA		MSSA
MSSA		MSSA
MSSA<br>*Pseudomonas aeruginosa*		MSSA<br>*Pseudomonas aeruginosa*
Pseudomonas aeruginosa		No recurrence
MSSA		No recurrence
MSSA		MSSA,<br>*Enterobacter species*
Pseudomonas aeruginosa		MSSA<br>*Pseudomonas aeruginosa*
MSSA		MSSA
Pseudomonas aeruginosa		*Pseudomonas aeruginosa*
MSSA		MSSA
Pseudomonas aeruginosa		*Serratia marcescens*
MSSA		MSSA
Enterobacter species		*Klebsiella* species
Pseudomonas aeruginosa		ESBL producing<br>*Pseudomonas aeruginosa*
Pseudomonas aeruginosa		*Pseudomonas aeruginosa*
MSSA		Recurrence (no organism identified)

Supplementary Figure 1

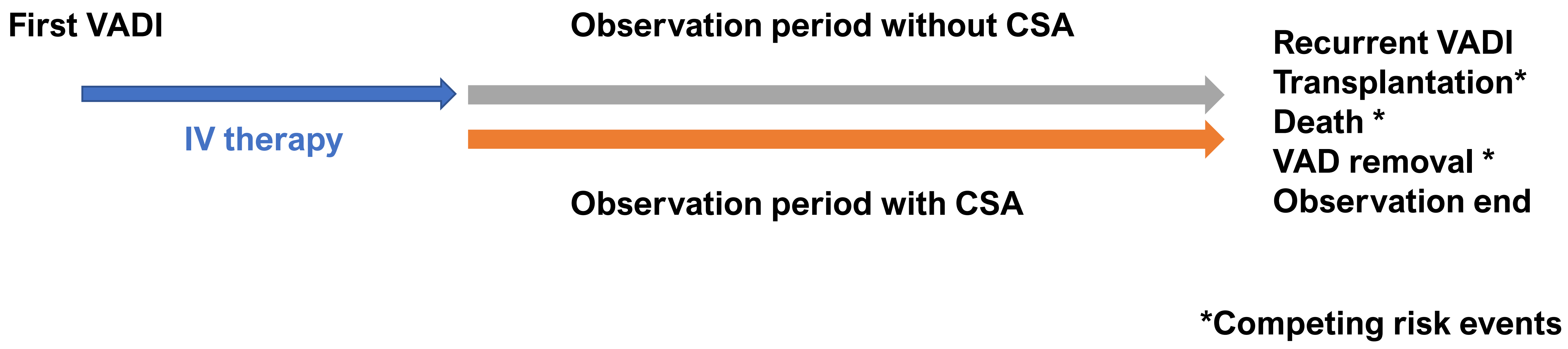

Supplementary Figure 2

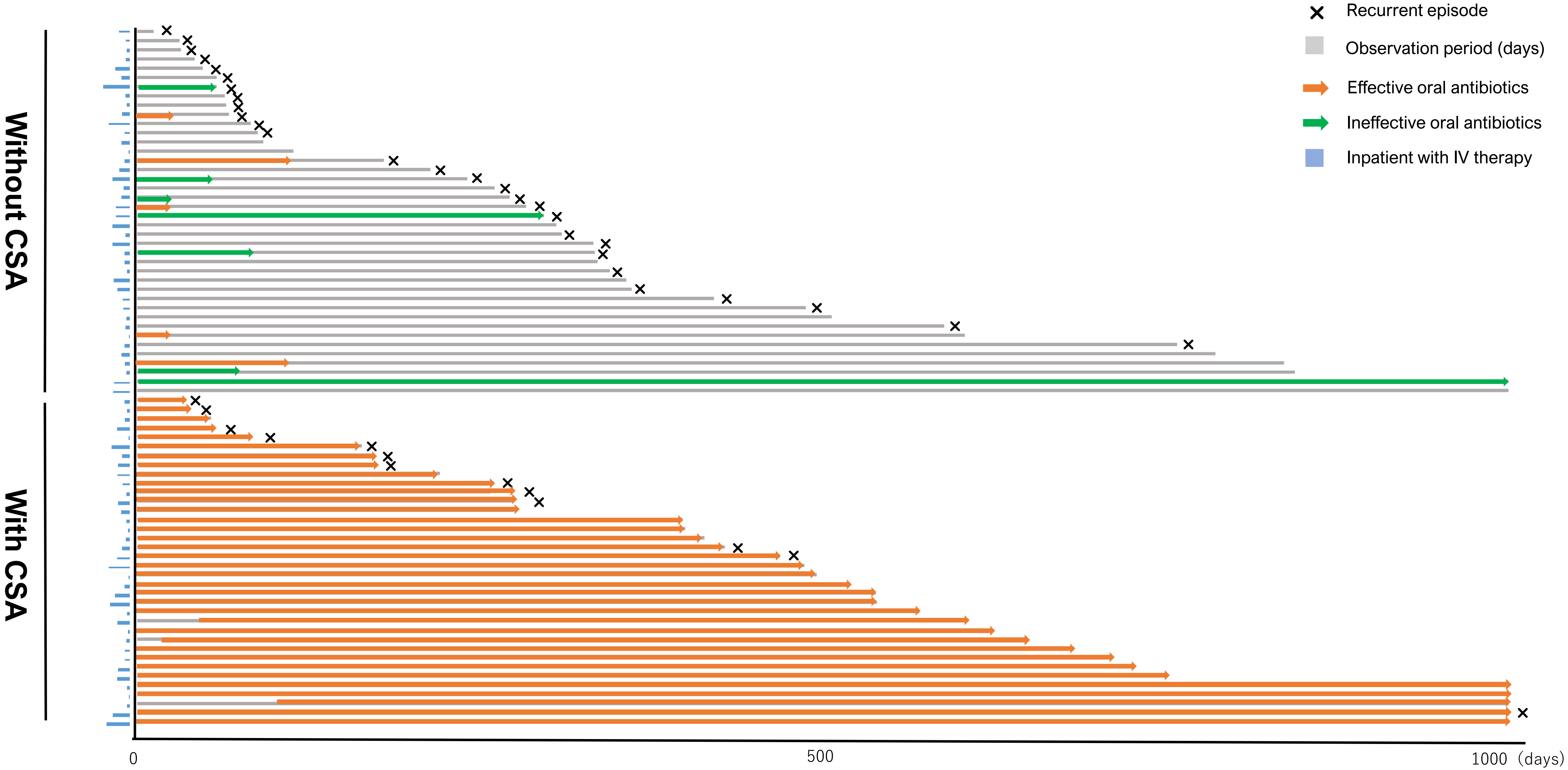

Supplementary Figure 3

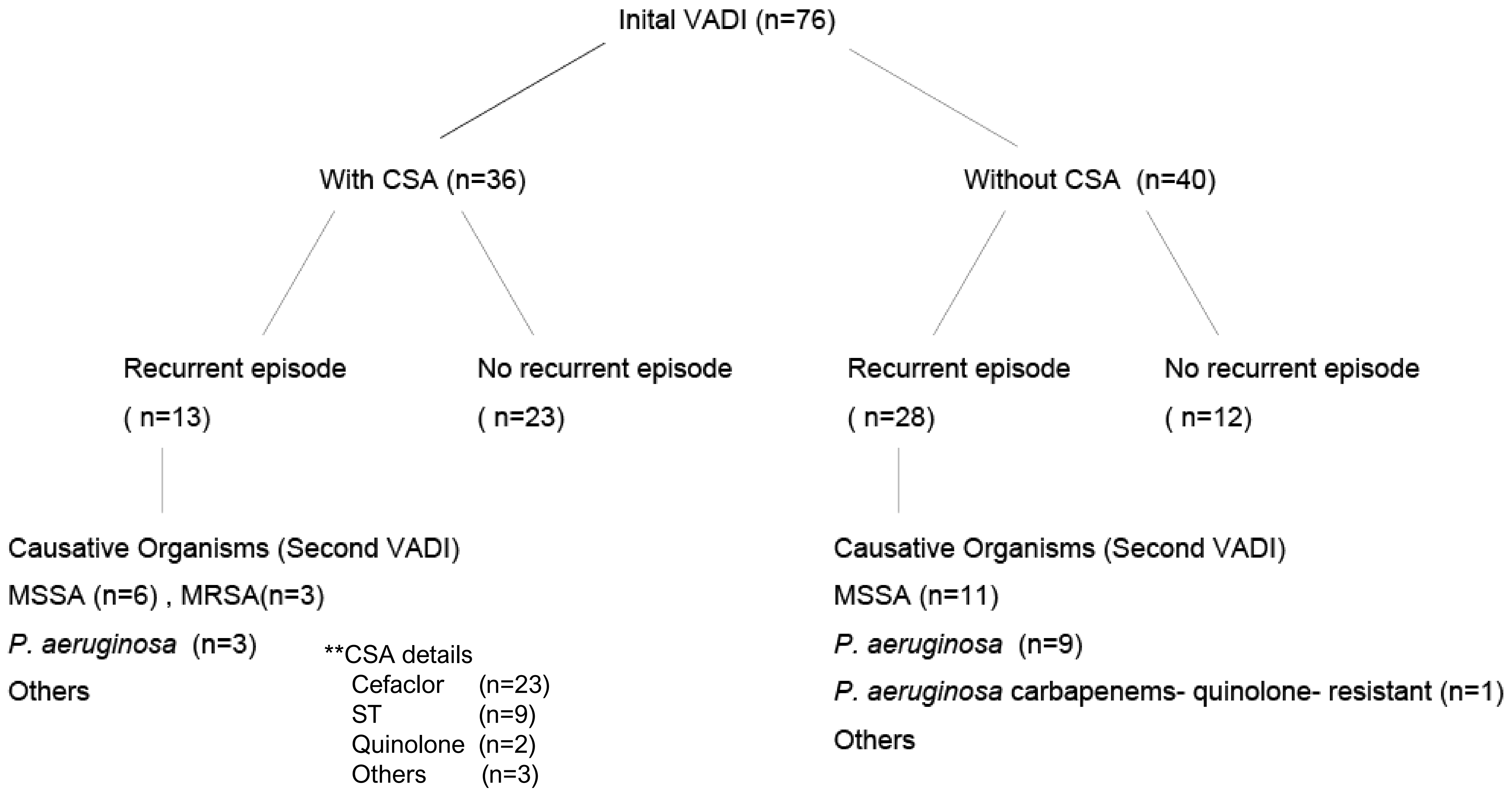

Supplement: Supplemental material — Fig. S1 to S3; Table S1. [file spectrum.00398-24-s0001.pdf]
